# The Effects of Bikram Yoga on Health: Critical Review and Clinical Trial Recommendations

**DOI:** 10.1155/2015/428427

**Published:** 2015-10-05

**Authors:** Zoe L. Hewett, Birinder S. Cheema, Kate L. Pumpa, Caroline A. Smith

**Affiliations:** ^1^School of Science and Health, Western Sydney University, Campbelltown Campus, Campbelltown, NSW 2560, Australia; ^2^The National Institute of Complementary Medicine, Western Sydney University, Campbelltown Campus, Campbelltown, NSW 2560, Australia; ^3^University of Canberra Research Institute for Sport and Exercise, University of Canberra, Bruce, ACT 2617, Australia

## Abstract

Bikram yoga is a style of *hatha* yoga involving a standarized series of *asanas* performed to an instructional dialogue in a heated environment (40.6°C, 40% humidity). Several studies evaluating the effect of Bikram yoga on health-related outcomes have been published over the past decade. However, to date, there are no comprehensive reviews of this research and there remains a lack of large-scale, robustly-designed randomised controlled trials (RCT) of Bikram yoga training. The purpose of this review is to contextualise and summarise trials that have evaluated the effects of Bikram yoga on health and to provide recommendations for future research. According to published literature, Bikram yoga has been shown to improve lower body strength, lower and upper body range of motion, and balance in healthy adults. Non-RCTs report that Bikram yoga may, in some populations, improve glucose tolerance, bone mineral density, blood lipid profile, arterial stiffness, mindfulness, and perceived stress. There is vast potential for further, improved research into the effects of Bikram yoga, particularly in unhealthy populations, to better understand intervention-related adaptations and their influence on the progression of chronic disease. Future research should adhere to CONSORT guidelines for better design and reporting to improve research quality in this field.

## 1. Introduction

**Figure 1:**
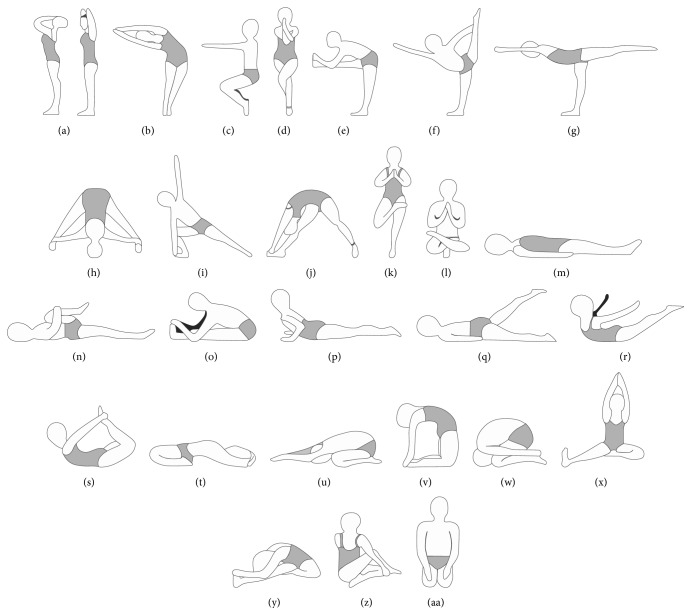
Bikram yoga series.

Bikram yoga is a popular, standardized system of* hatha* yoga developed by Choudhury [1], and, today, there are over 600 Bikram yoga studios worldwide [2]. Three factors together distinguish Bikram yoga from other forms of* hatha* yoga: (1) the set sequence of 26* asanas* and two breathing exercises (Figure 1), (2) the heated environment (40.6°C, 40% humidity), and (3) the instructional dialogue. Every 90 min class begins with standing* pranayama* (deep breathing) followed by the standing* asanas *(45–50 min, Figures 1(a)–1(l)). The standing sequence is followed by a 2 min* savasana* (supine relaxation, i.e., corpse pose, Figures 1(l) and 1(m)) and a sequence of floor* asanas* (35–40 min, Figures 1(n)–1(aa)). A 20-second* savasana* is taken between each* asana* in the floor series. Class finishes with a seated* kapalabhati* breathing exercise (i.e., quick, strong exhalations) and a final* savasana*. Choudhury suggests that the heated environment helps warm and prepare the body for movement and assists with removing impurities from the body [1].

Several studies have investigated the effects of Bikram yoga practice on health using various study designs [3–10]; however, to our knowledge, these studies have never been synthesized and critiqued and, accordingly, there is no consensus in the scientific literature regarding the effectiveness of Bikram yoga on health. Therefore, the purpose of this review is twofold: (1) to summarize studies that have investigated the effect of Bikram yoga practice on health-related outcomes and (2) to provide recommendations for the development of more robust trials and novel research questions to address the limitations of the existing literature.

## 2. Review of the Literature

Several studies have examined the chronic and acute effects of Bikram yoga practice in apparently healthy adults [3–5, 8, 9] and obese adults [9]. Health-related outcome measures that have been assessed include measures of physical fitness, cardiovascular disease risk factors, psychological health, pulmonary function, sleep quality, bone density, and metabolic cost. Only one trial reviewed used a randomised controlled trial (RCT) study design. A summary of these studies can be found in Table 1.

### 2.1. Physical Fitness

Physical fitness consists of five health-related domains (cardiovascular fitness, muscular endurance, muscular strength, flexibility, and body composition) and six skill-related domains (balance, reaction time, speed, agility, power, and coordination) [11]. The health-related components of physical fitness are particularly interesting as they are associated with better health status and QoL and lower risk of chronic diseases, disability, and mortality [12–14].

Four studies to date have evaluated the effect of Bikram yoga training on measures of health- and/or skill-related physical fitness [3–5, 8, 9]. Hart and Tracy [3, 4] examined the effects of an 8-week Bikram yoga intervention (3 classes/week) on body composition, flexibility, muscular strength and steadiness (neuromuscular control), cardiorespiratory fitness (VO_2max_), and balance in 21 apparently healthy adults. Thus far, this study is the only one to use an RCT design. Participants were randomised to a Bikram yoga group (*n* = 10) or control group (*n* = 11) and were instructed to maintain their current physical activity and dietary habits during the study. Upon completion of the 8-week intervention, the yoga group significantly increased lower body range of motion assessed by the standard sit-and-reach test as compared to the control group (*p* < 0.001). The yoga group significantly improved balance, as assessed by a single-leg balance test (*p* < 0.05), and significantly improved isometric dead-lift strength (*p* = 0.04) compared to the control group. Isometric maximal voluntary contraction evaluated via a load cell device at the knee joint significantly increased in the yoga group compared to the control group (*p* < 0.01), which showed a 10% decrease (*p* > 0.05). No changes were identified in upper body strength, namely, isometric handgrip strength (*p* = 0.30) or elbow flexor strength and steadiness. In support of these findings, significant improvements in sit-and-reach scores within and/or between groups after 3-3+ classes a week for eight weeks were reported in one uncontrolled trial [5] and one controlled trial [8] examining apparently healthy cohorts. Furthermore, the uncontrolled 8-week trial also reported significant improvements in single-leg balance (*p* < 0.01) as well as improved upper body range of motion as assessed by a total-body rotation test [5].

Improvements in range of motion and balance are unsurprising given the nature of* hatha* yoga, and these results are supported by previous research [15–17]. The* asanas* that emphasize trunk and hamstring flexibility in a Bikram class are held for anywhere between 10 and 60 seconds (for specific* asanas* see Figures 1(e)–1(h), 1(o), and 1(x)–1(z)) allowing for improved passive and active range of motion [18]. There is some evidence to suggest that locally applied moist heat increases active and passive range of motion of the muscles comparable to static stretching or an active warm-up [19, 20]. The heated environment in Bikram yoga (in addition to the physical activity of the yoga) may impose a similar effect on the tissues of the muscular system. The* asanas* that are performed on one leg for between 10 and 60 seconds (for specific* asanas* see Figures 1(c)–1(f) and 1(k)–1(l)) would likely contribute to the improvement in single-leg balance. An emphasis on* asanas* involving lower body strength and frequent isometric contraction of the quadriceps throughout the class (see Figures 1(c)–1(g), 1(i), 1(l), and 1(s)) likely explains improved lower body strength and unchanged upper body strength [17]. Future recommendations for assessing fitness outcomes include examining a cohort with musculoskeletal conditions that would benefit from improved muscular fitness and range of motion (e.g., cohorts with falls risk and low back pain). Although insignificant, greater changes in muscular steadiness were observed in participants with lower baseline values, supporting further investigation into the effects of Bikram yoga in sedentary individuals [3]. Additionally, improved single-leg balance in older adults after an Iyengar yoga intervention indicates that it may also be valuable to assess functional movement outcomes, for example, a 10-repetition maximum or Fallscreen assessment for falls risk [16, 21]. Improvements in flexibility, strength, and balance have a tremendous impact on QoL, especially in older adults, given that these aspects of physical fitness decline with age, are necessary for activities of daily living, and are associated with falls risk [22].

Body composition is a health-related component of fitness and excess adiposity is a cardiovascular disease risk (CVD) factor. To date, no Bikram yoga study has reported significant changes in adiposity or lean muscle mass when measured using dual-energy X-ray absorptiometry (DEXA) [4, 8, 9]. Tracy and Hart [4] reported a trend toward reduced body adiposity in the yoga group (*p* = 0.069), which, despite randomisation, had higher baseline adiposity (28.4 ± 6% versus 20.8 ± 8.1%, *p* = 0.03). Two controlled trials also reported no significant changes in body composition within or between groups after an 8-week Bikram yoga program (3x/week); however, older, obese participations showed a significant decrease in BMI from 34.3 ± 4.7 to 33.7 ± 4.9 (*p* < 0.05) [8, 9]. Though no significant changes have been observed to date, many factors contribute to alterations in body composition. Participants in these studies were asked not to change their current exercise and diet habits; however, energy intake and expenditure data during the trial were not collected. Therefore, it is difficult to ascertain whether or not external factors may have contributed to body composition measures at completion of the trial. Accounting for confounding factors and prescribing an effective intervention volume should be included in future RCT design examining the effects of Bikram yoga on body composition. Potential changes in body composition in certain populations (i.e., sedentary and unfit) after a Bikram yoga program could result from increased energy expenditure and/or increased muscle mass. Furthermore, intervention-related reductions in stress may improve regulation of hormones in the hypothalamic-pituitary-adrenal (HPA) axis, like cortisol, that are known to contribute to visceral adiposity [23, 24]. Previous research of other styles of* hatha* yoga reports acute and chronic intervention-related improvements to stress, inflammation, acute cortisol, leptin, and adiponectin [25–27]. Future Bikram yoga RCTs could examine additional markers including leptin, adiponectin, and cortisol to further investigate the effects of Bikram yoga on stress-related components of metabolism.

Despite the current lack of evidence for Bikram yoga as a tool to significantly improve body composition, acute data still lends valuable insight into the energy expenditure of and potential population-dependent (i.e., unfit and sedentary) body composition adaptations to a Bikram yoga class. A cross-sectional study of 24 apparently healthy adults of varying Bikram yoga experience used an environmental chamber and metabolic measurement cart (TrueOne, ParvoMedics) to examine the acute physiological adaptations to a single Bikram session performed to a standardised audio recording of a class [28]. The mean relative VO_2_ for the whole session was 9.5 ± 1.9 mL·kg^−1^·min^−1^ and the intensity of the class was 2.9 METS, with postures ranging from light to moderate intensity (<3.0 to 3.0–6.0 METS) over the class. Absolute energy expenditure ranged from 179 to 478 kcals per session (mean 286 ± 72 kcals). The more experienced group (>20 classes experience) reported significantly higher relative energy expenditure, predicted maximal heart rate, and sweat rate compared to the novice group (<20 classes experience). These findings are supported by similar trends in unpublished data presented at the 2013 Rocky Mountain ACSM Annual Meeting; however, the unpublished data reports higher values for energy expenditure and average intensity (333–459 kcals, 3.7 METS) over the whole class in experienced practitioners (>12 months experience, *n* = 19) [29]. Based on this acute data, and although it appears that Bikram yoga elicits a greater average in-session MET level compared to other forms of* hatha* yoga [30, 31], the prescribed 8-week interventions in the studies reviewed may be insufficient for weight loss considering that ACSM recommends building up to 250–300 minutes of moderate intensity aerobic exercise per week for weight loss and maintenance of long term weight loss [32]. Discrepancies in oxygen consumption values between these two current reports may be due to the experience level of the sample and possibly the method of gas analysis, although the latter is purely speculation. The initial* pranayama* exercise is performed by inhaling through the nose and exhaling through the mouth [1], which would be interrupted by wearing a nose clip. Breathing exercises aside, during most of the* asanas* in a* hatha* yoga class, the instruction is to breathe through the nose only [33, 34], and although there is no definitive data to suggest that submaximal measurements of oxygen consumption are affected by the type of gas analysis system (i.e., face mask or mouth tube and nose clip), it may be worth consideration in future oxygen consumption analysis of* hatha* yoga postures.

The only study examining aerobic capacity in a controlled manner reported no significant change in VO_2max_ over an 8-week period in apparently healthy adults (Balke treadmill test, TrueOne, ParvoMedics). This may explain why the aerobic capacity of Bikram practitioners is not significantly different from that of the general population [4, 7]. Research that has reported increased aerobic capacity after an 8-week Bikram yoga trial used a predictive VO_2max_ measure (1-mile walk), which is not as reliable as an actual VO_2max_ test. In-session VO_2_ data shows that the average intensity of a Bikram yoga session is light to moderate [28, 29], suggesting that the cardiovascular adaptation may be great enough to improve VO_2max_ in certain cohorts such as populations suffering from reduced cardiorespiratory fitness (e.g., sedentary individuals, asthmatics, and older adults), which is significant, as reduced cardiorespiratory fitness is considered an independent risk factor for cardiovascular disease [35]. Furthermore, intervention duration may have been too short to establish significant changes in cardiorespiratory fitness [32].

### 2.2. Cardiovascular Disease Risk Factors

World Health Organization reports that 38% of total deaths in the US are attributable to CVD and diabetes [36]. Several controlled trials have investigated the effect of Bikram yoga training on various CVD risk factors. One controlled trial examined the effects of an 8-week Bikram yoga program (3 classes/week) on arterial stiffness, measures of blood glucose regulation (HbA1c, fasting blood glucose, plasma insulin, and insulin resistance via homeostatic model assessment, i.e., HOMA-IR), blood lipids (total cholesterol, low density lipoprotein (LDL), high density lipoprotein (HDL), and triglycerides), blood pressure, and body composition in healthy young adults compared with older adults [8]. As mentioned previously, this trial did not report significant improvements in body composition. In the younger group, carotid artery compliance significantly increased (*p* < 0.05) and beta-stiffness significantly decreased (*p* < 0.05) compared to baseline. Longer interventions may not necessarily improve arterial stiffness in older adults, yet arterial stiffness is an independent risk factor for CVD [37]. Seeing as an appropriate prescription for Bikram yoga is still unclear; measuring arterial stiffness in this cohort using a longer Bikram yoga intervention with more frequent weekly sessions may be warranted even though there has been no observed change in this cohort to date [8].

Furthermore, HDL and total cholesterol decreased in young adults (*p* < 0.05), while LDL and total cholesterol decreased in older adults compared to baseline (*p* < 0.05). This finding in young adults is surprising considering that exercise generally contributes to HDL increases [38]. Fasting blood glucose and HbA1c did not change in either group; however, plasma insulin and HOMA-IR both significantly decreased in older adults only when compared to baseline (*p* < 0.01). Further investigation is essential to discern the likely mechanisms responsible for metabolic profile changes. Potential reasons for the significant changes include the stress-reducing effects of yoga [24], the heated environment [39], and unrecorded dietary changes. Scientific reviews report evidence of yoga improving diabetic symptoms and risk factors (including insulin sensitivity and glucose tolerance); however, more research is required to draw stronger conclusions [40, 41].

Brachial blood pressure (systolic and diastolic) did not decrease significantly, which was also the case in a previous 8-week* hatha* yoga trial in normotensive adults [8, 42]. Three additional studies have investigated the effect of Bikram yoga practice on resting heart rate and systolic and diastolic blood pressure [4, 5, 7, 8]. All four studies to date have been conducted with normotensive participants and, as expected, all reported no significant change over time [4, 5, 7, 8]. The cross-sectional study reported that, compared to novice students, regular Bikram yoga practitioners had lower mean blood pressure and resting heart rates than national US averages. It is possible that chronic Bikram yoga practice may help maintain healthy blood pressure values [7]. The light to moderate aerobic intensity over a longer duration (i.e., greater than 8 weeks) as well as more regular sessions (i.e., 3–7 days/week) may be enough in deconditioned and hypertensive participants to affect blood pressure [32, 43–45]. Another possible influence on blood pressure could be stress, and Bikram yoga has been shown to reduce perceived stress [5].

A more recent study by the same research group examined the effects of an 8-week Bikram yoga program (3 classes/week) on body composition and glucose tolerance in young, lean (BMI = 22.1 ± 2.1 kg/m^2^, 28 ± 7% body fat) and older, obese (BMI = 34.3 ± 4.7 kg/m^2^, 44 ± 6% body fat) sedentary participants [9]. Glucose tolerance significantly improved in older, obese participants (*p* < 0.05) but, as expected, not in young, lean participants. Authors suggest that this is likely due to the increased insulin resistance that occurs in obese populations but not in healthy populations. There were no significant changes in fasting plasma glucose over time in either group. In both controlled trials, participants were asked to continue with their normal diet and exercise routines; however, this data was not collected [8, 9]. Changes in diet and exercise would certainly influence glucose management outcomes, and exercise and diet data should be reported in future trials.

### 2.3. Pulmonary Function

Pulmonary function can be reduced as a consequence of aging [46] and chronic diseases, including asthma, emphysema, bronchitis, metabolic syndrome, and diabetes [47–50]. Reduced pulmonary function can have significant implications including reduced physical activity and associated chronic conditions including diabetes and CVD [51]. Aerobic exercise, inspiratory muscle training, diaphragmatic breathing training, and* hatha *yoga have been shown to improve pulmonary function outcomes in cohorts with reduced baseline values [52–55], whereas, in apparently healthy adults, no changes are seen after a 12-week aerobic exercise program [56].

A cross-sectional study examining measures of pulmonary function in 31 apparently healthy Bikram yoga practitioners reported no significant difference between those of limited experience (0.07 ± 0.06 years) and those of more experience (4.16 ± 2.8 years) [7]. There were no differences in height, weight, or age between the groups. When classified by gender, aerobic capacity (VO_2max_) of this cohort was classified as “good” according to ACSM [32]. It is unsurprising that pulmonary function and aerobic capacity were not different based on level of experience when compared to population norms; seeing as reduced pulmonary function (e.g., forced expired volume in one second) is seen only in those with pulmonary dysfunction [32]. Furthermore, because pulmonary function varies according to gender, it would be useful to have results presented for females and males separately to better understand the data. There was a weak but significant relationship between Bikram yoga experience and percent predicted forced vital capacity (*r* = 0.38, *p* < 0.05) and forced expired volume in one second (*r* = 0.37, *p* < 0.05). These results could have been influenced by the final breathing exercise in a Bikram yoga class, which uses the abdominal muscles to repeatedly exhale the breath at a moderate pace [1]. A review reports that, in apparently healthy adults, changes in pulmonary function in response to* hatha* yoga are related to initial fitness level and duration of* pranayama* exercises [57], which perhaps indicates that a Bikram yoga class does not have enough specific* pranayama* exercises to elicit a significant improvement in pulmonary function in healthy individuals. Future Bikram yoga RCTs are warranted, and in order to investigate further, future RCTs should address confounding factors, including ethnicity, gender, and smoking status, and should examine cohorts that have room to improve function, such as asthmatics or sedentary individuals.

### 2.4. Bone Mineral Density

Maintaining BMD throughout life is critical for health outcomes (osteoporosis, falls-related fractures) and QoL [58, 59]. In adults, the emphasis should be placed on nonpharmaceutical interventions such as nutrition, resistance training, and impact based exercise, for example, running, to minimize BMD loss and reduce the risk of fractures in later life. Given that Bikram yoga has been shown to significantly improve lower limb strength and single-leg balance it may reduce the risk of falls and fractures, similar to other* hatha* yoga interventions [3–5, 60]. As a suitable weight-bearing exercise for those who cannot engage in high-impact activities, it could be hypothesized that yoga may, via various pathways, maintain bone density in some populations [60].

A 5-year longitudinal study assessed BMD using DEXA in nine female Bikram yoga teachers (30–59 years) with 3+ years of regular teaching and practicing Bikram yoga [10, 61]. Those who were premenopausal at the 5-year follow-up showed mean increased BMD at the femoral neck (6.6%  ± 5.5%), total hip (2.0%  ± 3.8%), and lumbar spine (1%  ± 4.7%). Those who were postmenopausal at the 5-year follow-up showed a mean decrease in BMD at the femoral neck (−6.0%  ± 6.6%), total hip (−8.1%  ± 6.1%), and lumbar spine (−5.6%  ± 9.1%). The improved total hip BMD in the premenopausal group was significantly higher than the reduction in BMD reported by the postmenopausal group (*p* = 0.02). These findings are surprising in the premenopausal group; however, the results were as expected in the postmenopausal group due to the age-related decline in BMD. Possible confounding factors, such as whether or not participants engaged in additional exercise, altered nutritional intake or took certain medication during the 5-year longitudinal period were not reported. More rigorous RCTs that incorporate measures such as hormone fluctuations during menopause and changes in nutritional intake could further explain these findings.

### 2.5. Sleep Quality

Quality of sleep is affected by several factors including stress, hormonal imbalances, and obesity [62]. Although exact mechanisms are unknown, both exercise and yoga reportedly encourage healthy sleep patterns [63–65]. An uncontrolled, observational study examined the acute effects of Bikram yoga practice on the sleep architecture (structure and pattern of sleep) of 13 apparently healthy males and females aged between 18–45 years [6]. Participants were asked to partake in 2–12 Bikram yoga classes over a period of 14 days to compare sleep patterns (wireless headband and self-report) on the days when participants attended class compared with days when participants did not. Total sleep time, sleep latency (time it takes to fall asleep), and stages of sleep (i.e., REM cycles) were unchanged between practice and nonpractice days. On days when participants attended class, the time to return to sleep after naturally occurring nocturnal awakenings was reduced significantly (*p* < 0.03). No study has examined the effect of Bikram yoga on hormones (e.g., melatonin, serotonin, and dopamine), which may influence sleep patterns, but this finding could also be influenced by the stress-reducing effect of Bikram yoga that has been observed in a separate study [5]. Future research should consider using a cohort prone to sleep disturbances.

### 2.6. Psychological Adaptations

Chronic stress is now widely acknowledged as being associated with CVD among a wide range of other chronic conditions. Stress contributes to systemic inflammation via two main pathways, the HPA axis and the sympathetic nervous system [66, 67]. It has been hypothesized that* hatha* yoga practice may attenuate HPA axis and sympathetic hyperactivity and the associated physiological, inflammatory response, which could lead to a reduction in stress- and inflammation-related illness [26].

An uncontrolled trial examined the effectiveness of 8 weeks of Bikram yoga (3+ classes/week) on mindfulness, perceived stress, and physical fitness in 51 apparently healthy men and women [5]. Participants (20–54 y) had varying physical activity levels at baseline. At the completion of the trial, mindfulness, evaluated by the Five-Facet Mindfulness Questionnaire (FFMQ), and perceived stress, evaluated by the Perceived Stress Scale (PSS), significantly improved (*p* < 0.01). Mindfulness was negatively and significantly correlated with perceived stress (*r* = −0.43, *p* < 0.05) and with resting heart rate (*r* = 0.30, *p* < 0.01).* Hatha* yoga offers a “meditation through movement” opportunity much like the practice of Tai Chi [68], which may contribute to the increased mindfulness and reduction in perceived stress. Although it is hard to know what is responsible for these changes,* hatha* yoga incorporates physical activity as well as relaxation in the same class and encourages participants to keep the mind present with the movement of the breath and the body in different* asanas*. These qualities may encourage mindfulness, increase vagal tone, and reduce perceived stress, and they present opportunities for more robust research examining stress reduction and associated health risks.

Despite major limitations in this study's design, these preliminary findings provide a starting point for understanding Bikram yoga's relationship to stress and, potentially, stress-related illnesses and chronic disease. Further recommendations for the investigation of psychological outcomes should include the collection of more self-report data as well as physiological outcomes with a known relationship to psychological stress, including heart rate variability (HRV), arterial stiffness, interleukin-6 (IL-6), CRP, and cortisol [66, 69, 70]. HRV has been shown to improve with some* hatha* yoga interventions [42, 71]. HRV also relates to cardiovascular outcomes and may address the relationship between psychological stress and chronic disease. Investigating intervention-related behaviour change may be noteworthy to assess the effects of yoga practice on lifestyle. The findings from this preliminary research warrant further investigation in RCT format considering the contribution stress has towards health and well-being.

### 2.7. Adherence

Adherence data is important to assess the feasibility of any physical activity intervention. It is also crucial in explaining outcome measures that change, or remain the same, as a result of an intervention. For example, a participant who attends one exercise session per week will likely not improve health measures to the same degree that a participant attending four sessions would [72]. Only two of the Bikram yoga studies reviewed reported adherence data. The RCT reported a retention rate of 48% in the yoga group [4], with an average attendance of 22.5 classes in eight weeks, and dropouts were attributed to scheduling and dissatisfaction with the intervention [4]. An uncontrolled trial studying mindfulness reported a 64% retention rate, with average attendance of 28.6 classes in eight weeks, and the majority of dropouts were due to time commitments [5].

### 2.8. Adverse Events

Two trials reported no adverse events [8, 9], and all other trials failed to report on adverse events at all; however, Bikram yoga, like any exercise prescription, may be unsafe for certain individuals and medical clearance is advised for at risk individuals [8, 9, 32, 73]. Individual cases of adverse events have been reported in three separate case studies [73–75]. A letter to the editor of the American Journal of Psychiatry was submitted in 2007 reporting a case study of a 33-year-old man with a history of brief hallucinogen-induced psychosis who experienced a psychotic episode while participating in the Bikram yoga teacher training. The subject reported feeling dehydrated and eating poorly and was lacking sleep leading up to the episode. Hospital tests (brain MRI, EEG, and urine toxicology) all came back normal. The authors suggest that despite reported benefits for some individuals more intensive styles of yoga (i.e., Bikram yoga) may not be suitable for certain individuals such as those who are psychosis-prone.

The second case study reported on a 34-year-old woman who presented to hospital with breathlessness, muscle cramps, nausea, and general malaise from drinking 3.5 liters of water after her first Bikram yoga class [75]. Initial testing (blood gas on air) showed severe hyponatremia and respiratory alkalosis. After 5 days in hospital a full recovery was made and the woman was discharged. Bikram yoga instructors should be aware of the risk factors, signs, and symptoms of hyponatremia given the extreme hot and humid conditions in the class, and they should be able to make sound recommendations for rehydration including drinking to one's thirst [76].

A third case study reported on a healthy 53-year-old man with no CVD risk factors who experienced acute coronary syndrome during a Bikram yoga class [74]. The man was admitted to hospital and received surgical intervention before being discharged 4 days later. Males aged ≥45 years and women aged ≥55 are at increased risk of heart disease [32] and practitioners in this age group should exercise caution, potentially obtaining doctor's clearance, when starting Bikram yoga, especially if coming from a sedentary lifestyle.

Although these are isolated events, it is of great importance to understand the potential risk of participation in any form of exercise for different populations, especially exercise such as Bikram yoga, which has not yet been thoroughly examined with repeated, robust research. Future research should disclose all adverse events and employ satisfactory preintervention screenings and medical supervision when required, taking into account the potential risk of Bikram yoga for the given sample population.

## 3. Future Recommendations

It appears that Bikram yoga training improves aspects of physical fitness (range of motion, single-leg balance, and strength), which can be logically explained by the nature of Bikram yoga, a style of* hatha* yoga. A deeper, scientific examination is required to determine the effect of Bikram yoga on cardiovascular outcomes (cholesterol, fasting blood glucose, arterial stiffness, and carotid artery compliance), bone mineral density, sleep quality, pulmonary function, and psychological health (perceived stress and mindfulness). The greatest improvement to the current body of scientific knowledge of Bikram yoga lies in future application of robust research techniques.

Only one study in this review used an RCT design to examine the effects of Bikram yoga. Fundamentally, subsequent Bikram yoga studies should adopt an RCT design in order to add significant contributions to the discussion on the health benefits derived from exercise interventions. Greater adherence to reporting standards, such as CONSORT, may improve reporting and assessment of study quality from current standards and would address the pervasive study limitations present in the current body of research including small sample sizes, lack of randomization, confounding variables, and little mention of the occurrence or lack of adverse events, limitations, and compliance [77].

Bikram yoga appears to be an alternative to traditional exercise modalities that may have favourable effects on metabolic markers including blood lipids, insulin resistance, and glucose tolerance. This has significant clinical implications for individuals who wish to address dyslipidemia and glucose management by exploring nonpharmaceutical options. Additionally, those who are unable or unwilling to participate in traditional aerobic exercise and strength training may be able to improve glucose and cholesterol management with Bikram yoga training. In order to maximize the effect of these findings, trial duration and session frequency need to be addressed in future research. This is more complex than adhering to CONSORT or ACSM guidelines.* Hatha* yoga is a holistic and often variable practice making research hard to quantify and compare to other forms of exercise [78]. Further understanding of the acute adaptations to Bikram yoga is crucial to determine whether or not this form of exercise is an effective intervention compared to current ACSM guidelines for exercise and disease management and prevention. This would allow for a comparison between Bikram yoga and other exercise modalities. The metabolic cost data reported in this review is very new information and could be used in future research to determine a specific Bikram yoga prescription, knowing that the prescription will vary depending on the desired outcome measure. Additional acute data, for example, blood glucose before, during, and after a session, would also contribute to the development of Bikram yoga prescription guidelines for specific outcome measures.

Finally, most of the current studies have examined apparently healthy adults. Unsurprisingly, health-related outcomes, for example, resting heart rate and blood pressure, remain unchanged in healthy participants. It is hard to determine the effects of an exercise intervention in cohorts whose indicators of health and well-being are within healthy ranges at baseline. Significant findings in one study to the authors' knowledge that has examined the effects of Bikram yoga on the unhealthy (obese) individual strengthen the recommendation to examine cardiometabolic adaptations in unhealthy populations [9]. Physiological adaptations should be examined in cohorts with physiological imbalances, such as dyslipidemia, hypertension, and insulin resistance, to better understand intervention-related adaptations and their influence on the progression of chronic disease.

This review describes the available Bikram yoga literature in order to better understand the effects of this form of exercise and to suggest ways to improve future research in this field. Topics that have not yet been investigated include the effects of Bikram yoga on the physiological markers of stress, cognition, depression and anxiety, inflammation, QoL, and behaviour change. Additionally, further investigation into the acute effects of Bikram yoga practice would deepen the understanding of the physiological adaptations. Continued research will greatly improve the scientific understanding of Bikram yoga practice, which will help determine whether or not it can be considered another tool to address the concerning, growing prevalence of chronic disease and stress-related illness.

## Figures and Tables

**Table 1:** Characteristics of Bikram trials reviewed.

Study identification	Country	Sample size	Population		Sex (M/F)	Mean age (years)	Treatments	Control conditions	Trial duration (weeks)	Outcome measures	Main findings
			Major inclusion criteria	Major exclusion criteria							
Randomized controlled trial											
Tracy and Hart, 2013 [4]; Hart and Tracy, 2008 [3]	USA	21	Apparently healthy, relatively sedentary (<2 hours/week purposeful exercise <moderate intensity, one active subject in the yoga group and one in the control group)	Medical condition or medication that could influence participation or dependent measures and yoga practice within 4 months prior to study	M/F	27	Bikram's beginner's yoga class (room heated to 40°C, 40% humidity, same 26 *asanas* and breathing exercises each class), 90 min/class, 3 classes/week. Average attendance 22.5 classes	No treatment	8	Physical strength (isometric deadlift, hand grip strength, MVC of knee extensors/elbow flexors, concentric/eccentric steadiness), functional fitness (sit-and-reach and shoulder flexibility, single-leg balance), cardiovascular fitness (VO2max, resting BP), body composition (fat mass and lean body mass using DEXA)	Increased isometric deadlift strength (*p* = 0.04 between groups), increased knee extensor MVC within and between groups (*p* < 0.05, *p* < 0.01), increased balance yoga group (*p* < 0.05), increased back/hamstring flexibility (*p* < 0.001 between groups), trend towards significance for shoulder flexibility and for decreased fat mass in yoga group (*p* = 0.069)

Controlled trials											
Hunter et al., 2013 [9]	USA	Young = 14 Older = 15	Sedentary (<2 days/week physical activity for past 6 months), lean participants BMI 18.5–24.9 and obese participants BMI >30	Smoking within last 6 months, uncontrolled diabetes, personal history of stroke, prior myocardial infarction, and known coronary heart disease	M/F	Young = 32 Older = 46	Bikram's beginner's yoga class (room heated to 40°C, 40% humidity, same 26 *asanas* and breathing exercises each class), 90 min/class, 3 classes/week	No nonyoga control	8	Glucose tolerance (75 g GTT, FPG) body composition (fat mass and lean body mass using DEXA, BMI, body mass)	Increased glucose tolerance in obese subjects (*p* < 0.05), decreased body mass (*p* < 0.05) and BMI (*p* < 0.05) in obese subjects
Hunter et al., 2013 [8]	USA	Young = 24 Older = 18	Sedentary (no habitual activity over last 6 months), young participants aged 18–39 and older participants aged 40–70	Pregnancy, uncontrolled hypertension, infection within last 4 weeks, renal disease, adrenal or endocrine tumours, prior myocardial infarction, known coronary heart disease, chronic heart failure, personal history of stroke or cardiac arrhythmias, diabetes, heat intolerance, and cardiovascular or hormone replacement therapy medications	M/F	Young = 30 Older = 53	Bikram's beginner's yoga class (room heated to 40°C, 40% humidity, same 26 *asanas* and breathing exercises each class), 90 min/class, 3 classes/week	No nonyoga control	8	Arterial stiffness (carotid artery compliance, carotid pulse pressure), body composition (body mass, fat mass using DEXA), blood glucose measures (HbA1c, FBG, plasma insulin, HOMA-IR), cardiovascular health (total, LDL, and HDL cholesterol, triglycerides, BP), flexibility (sit-and-reach)	Increased carotid artery compliance in young adults (*p* < 0.05), decreased arterial stiffness in young adults (*p* < 0.05), decreased plasma insulin and HOMA-IR in older group (*p* < 0.01), decreased total and LDL cholesterol in older group (*p* < 0.05), decreased total and HDL cholesterol in young group (*p* < 0.05), increased flexibility in both groups (*p* < 0.01)

Uncontrolled trials											
Hewett et al., 2011 [5]	USA	51	NR	Bikram yoga practice at all in past 3 months and long term within last 2 years and medical conditions that did not pass medical clearance for participation	M/F	32	Bikram's beginner's yoga class (room heated to 40°C, 40% humidity, same 26 *asanas* and breathing exercises each class), 90 min/class, ≥3 classes/week. Average attendance 28.6 classes	No control	8	Psychological health (perceived stress, mindfulness), cardiovascular fitness (predicted VO2max, RHR), physical fitness (sit-and-reach and shoulder flexibility, single-leg balance)	Increased mindfulness (*p* < 0.01, *d* = 0.89), perceived stress (*p* < 0.01, *d* = −0.79), predicted VO2 (*p* < 0.01, *d* = 0.24), flexibility (*p* < 0.01, *d* = 0.63), balance (*p* < 0.01, *d* = 0.53), correlation between mindfulness and perceived stress (*r* = −0.43, *p* < 0.01), and mindfulness and resting heart rate (*p* = −0.30, *p* < 0.04)
Kudesia and Bianchi, 2012 [6]	USA	13	Apparently healthy, were planning to start or already practicing Bikram yoga	Medical problems or medications that might interfere with sleep monitor's algorithm (e.g., epilepsy)	M/F	35	Bikram's beginner's yoga class (room heated to 40°C, 40% humidity, same 26 *asanas* and breathing exercises each class), 90 min/class, 2–12 classes/14 days	N/A	2	Sleep architecture (time spent in each sleep-wake stage, duration of awakenings)	Decreased duration of awakenings on days of Bikram yoga practice (more rapid return to sleep after nocturnal awakenings)

Longitudinal study											
Sangiorgio et al., 2014 [10]	USA	9	Female (30–59 y), certified Bikram yoga instructors, practicing minimum 3 years, good physical health	NR	F	51 (at 5-year follow-up)	Continued practice of Bikram's beginner's yoga class (room heated to 40°C, 40% humidity, same 26 *asanas* and breathing exercises each class), 90 min/class, 3+ classes/week for 5 years. Continued teaching of Bikram yoga during 5-year period.	N/A	5 years	Bone mineral density (using DEXA to measure at the spine and hip, and total body)	Premenopausal women at follow-up showed mean increased BMD at the femoral neck (6.6% ± 5.5%), total hip (2.0% ± 3.8%), and lumbar spine (1% ± 4.7%). Postmenopausal women at follow-up showed mean decrease in BMD at the femoral neck (−6.0% ± 6.6%), total hip (−8.1% ± 6.1%), and lumbar spine (−5.6% ± 9.1%).

Cross-sectional and acute studies											
Pate and Buono, 2014 [28]	USA	26	Healthy adults (18–57 y), current Bikram practitioners with varying levels of experience (<20 classes and >20 classes)	Positive responses on PAR-Q and pregnancy	M/F	33	N/A	N/A	N/A	Acute response to Bikram yoga session in temperature controlled chamber (40°C, 40% humidity) including metabolic (VO2), cardiovascular (HR), and sweat rate response	Average overall VO2 9.5 mL/kg/min, average overall intensity 2.9 METS, average overall EE/session 286 kcal (179–478), and higher relative EE for more experienced practitioners
Abel et al., 2012 [7]	USA	LE = 17 HE = 14	Apparently healthy adults	Signs or symptoms of heart, pulmonary, or metabolic disease	M/F	LE = 44 HE = 38	N/A	N/A	N/A	Pulmonary function (FVC, FEV1, FVC/FEV1, PEFR, MVV), cardiovascular fitness (VO2max, RHR, BP)	Weak correlation of Bikram experience with FEV1 (*r* = 0.37) and with % predicted FVC (*r* = 0.38)
Fritz et al. 2013 [29]	USA	19	Current Bikram yoga practitioners attending ≥2 classes/week for at least 1 year	NR	M/F	30	N/A	N/A	N/A	Acute response to Bikram yoga session in temperature controlled chamber (40°C, 40% humidity) including metabolic (VO2), cardiovascular (HR), and thermal (internal temperature) response and RPE	Average VO2 13 mL/kg/min, average RPE 4.5, average HR 134 BPM, average overall intensity 3.7 METs, overall EE 333–459 kcal, and elevated core temperature within safe range (max 101.6°F)

BMI, body mass index; BP, blood pressure; BPM, beats per minute; CV, coefficient of variation; DXA, dual-energy X-ray absorptiometry; EE, energy expenditure; EEG, electroencephalogram; F, female; FEV1, forced expiratory volume in one second; FPG, fasting plasma glucose; FVC, forced vital capacity; GTT, glucose tolerance test; HbA1c, haemoglobin A1c; HE, high experience; HOMA-IR, homeostasis model of assessment of insulin resistance; HR, heart rate; LE, low experience; M, male; MRI, magnetic resonance imaging; MVC, maximal voluntary contraction; MVV, maximum voluntary ventilation; NR, not reported; PAR-Q, physical activity readiness questionnaire; PEFR, peak expiratory flow rate; RHR, resting heart rate; RPE, rate of perceived exertion; VO2, volume of oxygen uptake.

## References

[1] Choudhury B. (2007). *Bikram Yoga*.

[2] http://www.bikramyoga.com/studioListing.php.

[3] Hart C. E. F., Tracy B. L. (2008). Yoga as steadiness training: effects on motor variability in young adults. *Journal of Strength and Conditioning Research*.

[4] Tracy B. L., Hart C. E. F. (2013). Bikram yoga training and physical fitness in healthy young adults. *Journal of Strength and Conditioning Research*.

[5] Hewett Z. L., Ransdell L. B., Gao Y., Petlichkoff L. M., Lucas S. (2011). An examination of the effectiveness of an 8-week bikram yoga program on mindfulness, perceived stress, and physical fitness. *Journal of Exercise Science and Fitness*.

[6] Kudesia R. S., Bianchi M. T. (2012). Decreased nocturnal awakenings in young adults performing bikram yoga: a low-constraint home sleep monitoring study. *ISRN Neurology*.

[7] Abel A. N., Lloyd L. K., Williams J. S., Miller B. K. (2012). Physiological characteristics of long-term Bikram yoga practitioners. *Journal of Exercise Physiology Online*.

[8] Hunter S. D., Dhindsa M. S., Cunningham E. (2013). The effect of bikram yoga on arterial stiffness in young and older adults. *Journal of Alternative and Complementary Medicine*.

[9] Hunter S. D., Dhindsa M., Cunningham E., Tarumi T., Alkatan M., Tanaka H. (2013). Improvements in glucose tolerance with Bikram yoga in older obese adults: a pilot study. *Journal of Bodywork and Movement Therapies*.

[10] Sangiorgio S. N., Mukherjee A. K., Lau N. W., Mukherjee A., Mukhopadhyay P., Ebramzadeh E. (2014). Optimization of physical activity as a countermeasure of bone loss: a 5-year study of bikram yoga practice in females. *Health*.

[11] Hoeger W., Hoeger S. (2010). *Principles and Labs for Fitness and Wellness*.

[12] Center of Disease Control and Prevention Physical activity and health. http://www.cdc.gov/physicalactivity/everyone/health/index.html.

[13] Chien M.-Y., Kuo H.-K., Wu Y.-T. (2010). Sarcopenia, cardiopulmonary fitness, and physical disability in community-dwelling elderly people. *Physical Therapy*.

[14] Leś A., Gaworska M. (2011). Quality of life and functional fitness of the elderly. *Biomedical Human Kinetics*.

[15] Lau C., Yu R., Woo J. (2015). Effects of a 12-week hatha yoga intervention on cardiorespiratory endurance, muscular strength and endurance, and flexibility in Hong Kong Chinese adults: a controlled clinical trial. *Evidence-Based Complementary and Alternative Medicine*.

[16] Tiedemann A., O'Rourke S., Sesto R., Sherrington C. (2013). A 12-week Iyengar yoga program improved balance and mobility in older community-dwelling people: a pilot randomized controlled trial. *The Journals of Gerontology Series A: Biological Sciences and Medical Sciences*.

[17] Tran M. D., Holly R. G., Lashbrook J., Amsterdam E. A. (2001). Effects of hatha yoga practice on the health-related aspects of physical fitness. *Preventive Cardiology*.

[18] Roberts J. M., Wilson K. (1999). Effect of stretching duration on active and passive range of motion in the lower extremity. *British Journal of Sports Medicine*.

[19] Robertson V. J., Ward A. R., Jung P. (2005). The effect of heat on tissue extensibility: a comparison of deep and superficial heating. *Archives of Physical Medicine and Rehabilitation*.

[20] Knight C. A., Rutledge C. R., Cox M. E., Acosta M., Hall S. J. (2001). Effect of superficial heat, deep heat, and active exercise warm-up on the extensibility of the plantar flexors. *Physical Therapy*.

[21] Lord S. R., Menz H. B., Tiedemann A. (2003). A physiological profile approach to falls risk assessment and prevention. *Physical Therapy*.

[22] Ehrman J., Gordon P., Visich P., Keteyian S. (2013). *Clinical Exercise Physiology*.

[23] Kyrou I., Tsigos C. (2009). Stress hormones: physiological stress and regulation of metabolism. *Current Opinion in Pharmacology*.

[24] Rosmond R. (2005). Role of stress in the pathogenesis of the metabolic syndrome. *Psychoneuroendocrinology*.

[25] Kiecolt-Glaser J. K., Christian L. M., Andridge R. (2012). Adiponectin, leptin, and yoga practice. *Physiology & Behavior*.

[26] Kiecolt-Glaser J. K., Christian L., Preston H. (2010). Stress, inflammation, and yoga practice. *Psychosomatic Medicine*.

[27] Michalsen A., Grossman P., Acil A. (2005). Rapid stress reduction and anxiolysis among distressed women as a consequence of a three-month intensive yoga program. *Medical Science Monitor*.

[28] Pate J. L., Buono M. J. (2014). The physiological responses to Bikram yoga in novice and experienced practitioners. *Alternative Therapies in Health and Medicine*.

[29] Fritz M. L., Grossman A. M., Mukherjee A., Hunter S. D., Tracy B. L. Acute metabolic, cardiovascular, and thermal responses to a single session of Bikram yoga.

[30] Hagins M., Moore W., Rundle A. (2007). Does practicing hatha yoga satisfy recommendations for intensity of physical activity which improves and maintains health and cardiovascular fitness?. *BMC Complementary and Alternative Medicine*.

[31] Clay C. C., Lloyd L. K., Walker J. L., Sharp K. R., Pankey R. B. (2005). The metabolic cost of hatha yoga. *The Journal of Strength & Conditioning Research*.

[32] ACSM (2006). *ACSM's Guidelines for Exercise Testing and Prescription*.

[33] Hewitt J. (1983). *Complete Yoga Book*.

[34] Iyengar B. (1991). *Light on Yoga*.

[35] Franklin B. A., McCullough P. A. (2009). Cardiorespiratory fitness: an independent and additive marker of risk stratification and health outcomes. *Mayo Clinic Proceedings*.

[36] World Health Organization (2011). *Global Status Report on Noncommunicable Diseases 2010: Description of the Global Burden of NCDs, Their Risk Factors and Determinants*.

[37] Vlachopoulos C., Aznaouridis K., Stefanadis C. (2010). Prediction of cardiovascular events and all-cause mortality with arterial stiffness: a systematic review and meta-analysis. *Journal of the American College of Cardiology*.

[38] Ginsberg H. N. (2000). Nonpharmacologic management of low levels of high-density lipoprotein cholesterol. *The American Journal of Cardiology*.

[39] Gupte A. A., Bomhoff G. L., Swerdlow R. H., Geiger P. C. (2009). Heat treatment improves glucose tolerance and prevents skeletal muscle insulin resistance in rats fed a high-fat diet. *Diabetes*.

[40] Innes K. E., Vincent H. K. (2007). The influence of yoga-based programs on risk profiles in adults with type 2 diabetes mellitus: a systematic review. *Evidence-Based Complementary and Alternative Medicine*.

[41] Sharma M., Knowlden A. P. (2012). Role of yoga in preventing and controlling type 2 diabetes mellitus. *Journal of Evidence-Based Complementary & Alternative Medicine*.

[42] Papp M. E., Lindfors P., Storck N., Wändell P. E. (2013). Increased heart rate variability but no effect on blood pressure from 8 weeks of hatha yoga—a pilot study. *BMC Research Notes*.

[43] Pescatello L. S., Franklin B. A., Fagard R., Farquhar W. B., Kelley G. A., Ray C. A. (2004). Position stand: exercise and hypertension. *Medicine & Science in Sports & Exercise*.

[44] Cade W. T., Reeds D. N., Mondy K. E. (2010). Yoga lifestyle intervention reduces blood pressure in HIV-infected adults with cardiovascular disease risk factors. *HIV Medicine*.

[45] Pal A., Srivastava N., Tiwari S. (2011). Effect of yogic practices on lipid profile and body fat composition in patients of coronary artery disease. *Complementary Therapies in Medicine*.

[46] Stanojevic S., Wade A., Stocks J. (2008). Reference ranges for spirometry across all ages: a new approach. *American Journal of Respiratory and Critical Care Medicine*.

[47] Hesselbacher S. E., Ross R., Schabath M. B. (2011). Cross-sectional analysis of the utility of pulmonary function tests in predicting emphysema in ever-smokers. *International Journal of Environmental Research and Public Health*.

[48] Pelkonen M., Notkola I.-L., Nissinen A., Tukiainen H., Koskela H. (2006). Thirty-year cumulative incidence of chronic bronchitis and COPD in relation to 30-year pulmonary function and 40-year mortality: a follow-up in middle-aged rural men. *CHEST Journal*.

[49] Weiss S. T., Tosteson T. D., Segal M. R., Tager I. B., Redline S., Speizer F. E. (1992). Effects of asthma on pulmonary function in children: a longitudinal population-based study. *American Review of Respiratory Disease*.

[50] Yeh F., Dixon A. E., Marion S. (2011). Obesity in adults is associated with reduced lung function in metabolic syndrome and diabetes: the strong heart study. *Diabetes Care*.

[51] Adams R. J., Wilson D. H., Taylor A. W. (2006). Coexistent chronic conditions and asthma quality of life: a population-based study. *Chest*.

[52] Huang C.-H., Yang G.-G., Wu Y.-T., Lee C.-W. (2011). Comparison of inspiratory muscle strength training effects between older subjects with and without chronic obstructive pulmonary disease. *Journal of the Formosan Medical Association*.

[53] Shaw I., Shaw B. S., Brown G. A. (2010). Role of diaphragmatic breathing and aerobic exercise in improving pulmonary function and maximal oxygen consumption in asthmatics. *Science and Sports*.

[54] Vempati R., Bijlani R., Deepak K. K. (2009). The efficacy of a comprehensive lifestyle modification programme based on yoga in the management of bronchial asthma: a randomized controlled trial. *BMC Pulmonary Medicine*.

[55] Spruit M. A., Troosters T., Trappenburg J. C. A., Decramer M., Gosselink R. (2004). Exercise training during rehabilitation of patients with COPD: a current perspective. *Patient Education and Counseling*.

[56] Grisbrook T. L., Wallman K. E., Elliott C. M., Wood F. M., Edgar D. W., Reid S. L. (2012). The effect of exercise training on pulmonary function and aerobic capacity in adults with burn. *Burns*.

[57] Abel A. N., Lloyd L. K., Williams J. S. (2013). The effects of regular yoga practice on pulmonary function in healthy individuals: a literature review. *Journal of Alternative & Complementary Medicine*.

[58] Dennison E. M., Jameson K. A., Syddall H. E. (2010). Bone health and deterioration in quality of life among participants from the Hertfordshire cohort study. *Osteoporosis International*.

[59] Dennison E. M., Syddall H. E., Statham C., Aihie Sayer A., Cooper C. (2006). Relationships between SF-36 health profile and bone mineral density: the Hertfordshire Cohort Study. *Osteoporosis International*.

[60] Balk J., Bernardo L. M. (2011). Using yoga to promote bone health and reduce fracture risk in the geriatric population. *Topics in Geriatric Rehabilitation*.

[61] Mukherjee A., Mukherjee P., Rude R. R. (2010). Bikram yoga as a countermeasure of bone loss in women. *Chinese Medicine*.

[62] Gangwisch J. E., Malaspina D., Boden-Albala B., Heymsfield S. B. (2005). Inadequate sleep as a risk factor for obesity: analyses of the NHANES I. *Sleep*.

[63] Driver H. S., Taylor S. R. (2000). Exercise and sleep. *Sleep Medicine Reviews*.

[64] Taibi D. M., Vitiello M. V. (2011). A pilot study of gentle yoga for sleep disturbance in women with osteoarthritis. *Sleep Medicine*.

[65] Alexander G. K., Innes K. E., Selfe T. K., Brown C. J. (2013). ‘More than I expected’: perceived benefits of yoga practice among older adults at risk for cardiovascular disease. *Complementary Therapies in Medicine*.

[66] Foss B., Dyrstad S. M. (2011). Stress in obesity: cause or consequence?. *Medical Hypotheses*.

[67] Pasquali R., Vicennati V., Agostini A., Pagotto U. (2010). Glucocorticoids, stress and obesity. *Expert Review of Endocrinology & Metabolism*.

[68] Irwin M. R., Olmstead R. (2012). Mitigating cellular inflammation in older adults: a randomized controlled trial of Tai Chi Chih. *American Journal of Geriatric Psychiatry*.

[69] Sivasankaran S., Pollard-Quintner S., Sachdeva R., Pugeda J., Hoq S. M., Zarich S. W. (2006). The effect of a six-week program of yoga and meditation on brachial artery reactivity: do psychosocial interventions affect vascular tone?. *Clinical Cardiology*.

[70] Yadav R. K., Magan D., Mehta N., Sharma R., Mahapatra S. C. (2012). Efficacy of a short-term yoga-based lifestyle intervention in reducing stress and inflammation: preliminary results. *Journal of Alternative and Complementary Medicine*.

[71] Satyapriya M., Nagendra H. R., Nagarathna R., Padmalatha V. (2009). Effect of integrated yoga on stress and heart rate variability in pregnant women. *International Journal of Gynecology and Obstetrics*.

[72] Haskell W. L., Lee I.-M., Pate R. R. (2007). Physical activity and public health: updated recommendation for adults from the American College of Sports Medicine and the American Heart Association. *Circulation*.

[73] Lu J. S., Pierre J. M. (2007). Psychotic episode associated with Bikram yoga. *The American Journal of Psychiatry*.

[74] Ferrera C., Echavarría-Pinto M., Nuñez-Gil I., Alfonso F. (2014). Bikram yoga and acute myocardial infarction. *Journal of the American College of Cardiology*.

[75] Reynolds C. J., Cleaver B. J., Finlay S. E. (2012). Exercise associated hyponatraemia leading to tonic-clonic seizure. *BMJ Case Reports*.

[76] Hew-Butler T., Ayus J. C., Kipps C. (2008). Statement of the second international exercise-associated hyponatremia consensus development conference, New Zealand, 2007. *Clinical Journal of Sport Medicine*.

[77] Moher D., Schulz K. F., Altman D. G., Lepage L. (2001). The CONSORT statement: revised recommendations for improving the quality of reports of parallel-group randomised trials. *The Lancet*.

[78] Sherman K. J. (2012). Guidelines for developing yoga interventions for randomized trials. *Evidence-based Complementary and Alternative Medicine*.

